# Remarkable Case of Retention of the Placenta

**Published:** 1862-12

**Authors:** J. Drake Harper

**Affiliations:** Springfield, Ills.


### REMARKABLE CASE OF RETENTION OF THE PLACENTA.

By J. DRAKE HARPER, M. D., Springfield, Ills.

Mrs. B. gave birth to a fœtus of four months, on the 4th inst., late in the evening. I was sent for to remove the Placenta. On arriving, I found the cord had been ruptured within the os uteri, by a lady who was in attendance. I found the patient comfortable; no flooding; no after pains; pulse regular. On making an examination per vaginam I found the mouth of the womb almost entirely closed—not able to introduce the end of the index finger.

I gave her wine of Ergot, one drachm every half hour for six or eight hours. At the same time, directed warm water injections per the rectum and vagina, and cloths dipped in hot water to be applied over the hypogastric region.

On the morning of the 5th I called to see her. The remedies having effected nothing, I ordered a hip bath and a large dose of Castor Oil. After the oil had been taken a few hours I directed injections to be used again.

The evening of the same day I called again, finding my patient comfortable, oil having operated thoroughly; no flooding; no after pains; skin moist; pulse soft and regular. I made an examination per vaginam; the condition of the womb the same as at the former examination. The discharge from the vagina was of a sanious, watery nature, entirely void of smell.

That night I gave her three portions of Pulv. Doveri and small portions of Morphine, and Flax Seed Tea, to be used several times through the night.

On visiting my patient the following morning, she stated that she had had quite a good night's rest, perspired freely, and expressed herself as feeling able to get up and go to work.

This morning the indications were to let nature do its work, directing the use of Pennyroyal and Flax Seed tea occasionally through the day, and the use of warm water and milk injections, per vaginam, twice a day.

Believing "Conservative Medicine" to be safer than a "Meddlesome Midwife" in such cases, I directed the use of warm teas, and injections to be continued. I visited her regularly every day, until the evening of the tenth day after abortion. This evening I found her not so well; considerable fever; pulse 90; bowels same; tympanitis; slight tenderness over the region of the womb. Made an examination per vaginam. The mouth of the womb was still contracted, and hotter than natural, producing some pain in raising the womb upon the tip of the finger. Seeing interference became absolutely necessary to save my patient, I directed the use of a hip bath, injections to move the bowels, and hot fomentations to be applied over the abdomen. I also used a small quantity of Extract of Belladonna to the os tinceæ; and gave her—Doveri, grs. xxx; Sub. Mur. Hyd, grs. x; to be divided into four parts, one to be given every four hours, and Turpentine emulsion between the powders.

The next day at 12 o'clock M., I delivered the Placenta. She flooded a great deal before I arrived. Found the afterbirth hanging in the os uteri. On examination of the uterine surface of the Placenta it presented all the appearances of one recently expelled after the birth of a fœtus. There was no decomposition, hence there was no odor. The patient has entirely recovered.

